# New low-genus desingularizations of three Clifford tori and related characterizations

**DOI:** 10.1007/s10711-026-01100-2

**Published:** 2026-09-10

**Authors:** Nikolaos Kapouleas, David Wiygul

**Affiliations:** 1 Department of Mathematics, Brown University, Providence, RI 02912 USA; 2 Dipartimento di Matematica, Università degli studi di Trento, 38123 Povo, TN Italy

**Keywords:** Differential geometry, Minimal surfaces, Partial differential equations, 53A10

## Abstract

For each nonnegative integer *m* we construct in the round three-sphere a closed embedded minimal surface of genus 48m+25 which can be interpreted as a desingularization of the union of three Clifford tori intersecting pairwise orthogonally, along a total of six great circles. Each such surface is generated, under the action of a group of symmetries, by a disc with hexagonal boundary, all of whose sides are contained in great circles. We prove a uniqueness result for this disc, and, as a corollary, we characterize these surfaces. This characterization implies that similar surfaces we constructed for sufficiently high *m* by gluing methods, in an earlier article, coincide with the ones here. For low *m* the surfaces constructed here are new. Similarly, we prove uniqueness of the generating discs for one of two families constructed by Choe and Soret (namely the surfaces they call odd) and show that these surfaces also coincide, when of sufficiently high genus, with surfaces we have constructed by gluing.

## Introduction

A fundamental question is to classify the low-genus closed embedded minimal surfaces in the the round 3-sphere $\mathbb {S}^3$. The answer to this question is known only for genus zero by the uniqueness of the equatorial sphere proved by Almgren [1] using a method of Hopf, and for genus one by the uniqueness of the Clifford torus by the celebrated resolution of the Lawson conjecture by Brendle [3]. Note also that the equatorial sphere and the Cifford torus have the least area among closed embedded minimal surfaces in $\mathbb {S}^3$ by the celebrated resolution of the Willmore conjecture by Marques-Neves in [21], but no classification results are known at the moment for such surfaces of low area and genus g≥2.

The first such surfaces for any genus ≥2 were discovered by Lawson [20] via a tiling construction based on solving a Plateau problem to construct a fundamental domain of the surface. Modifying this approach, Karcher-Pinkall-Sterling discovered nine more such surfaces [14], Choe-Soret discovered more such surfaces desingularizing Clifford tori intersecting along two great circles [4], and Bai-Wang-Wang seven more such surfaces [2]. Karpukhin-Kusner-McGrath-Stern found such surfaces doubling the equatorial two-sphere by a variational approach based on maximizing the normalized first eigenvalue subject to appropriate symmetry and topological constraints [15]. Min-max constructions have also been successful in proving the existence of such surfaces, although so far they only provide limited control of their geometry and topology [16–18, 23, 24]. Constructions by PDE gluing methods have been very successful but so far they have been limited to high-genus surfaces [6–8, 11–13, 25, 26].

There are many cases of such surfaces constructed differently but resembling each other. In such cases they are often expected to be identical up to congruence, but not known to be so [8, Remark 5.23]. A natural proof would be by characterizing them by properties implied by their constructions. Unfortunately characterizations are known at the moment only for the Lawson surfaces [5, 9, 19].

Motivated partially from the above we construct in this article a new family of closed embedded minimal surfaces in $\mathbb {S}^3$ which includes low-genus examples not known before. The surfaces we construct desingularize three Clifford tori intersecting along six great circles and therefore have area less than $6\pi ^2$. Moreover we prove a characterization which implies that in the high-genus case they are identical to some of the surfaces we constructed in [11] by PDE gluing methods. We characterize similarly the surfaces in a family discovered by Choe-Soret in [4], and we conclude that they are identical to some other of the surfaces in [11]. We proceed to describe these results.

In [4] J. Choe and M. Soret constructed in the round 3-sphere $\mathbb {S}^3$ for each integer k≥2 two families, $\{T^o_{k,2km}\}_{m=1}^\infty$ and $\{T^e_{k,2km}\}_{m=2}^\infty$, of closed embedded minimal surfaces which can be regarded as desingularizations of the union of a maximally symmetric configuration $\textbf{T}[k]$ of *k* Clifford tori intersecting transversely along a pair of great circles at distance π/2. The surfaces $T^o_{k,2km}$ and $T^e_{k,2km}$ both have genus 1+4(k-1)km. Choe and Soret call these surfaces, respectively, odd and even, in view of their symmetries. The construction of the odd surfaces is reminiscent of Lawson’s tiling construction [20]; namely, Choe and Soret solve a certain Plateau problem with piecewise geodesic boundary in $\mathbb {S}^3$, and they then extend the solution to a closed embedded minimal surface by applying the group generated by the reflections through the great circles containing the boundary of the solution. The even surfaces are instead constructed by applying a different group of symmetries to the solution to a partially free boundary value problem, subject to a certain symmetry condition, but we will not discuss them further in this article.

In [11] we applied gluing methods to construct many closed embedded minimal surfaces admitting the same interpretation (as desingularizations of $\bigcup \textbf{T}[k]$) and also examples which desingularize rather the union of the collection $\textbf{T}[k,1]$ consisting of the Clifford tori in the preceding collection $\textbf{T}[k]$ together with the additional Clifford torus equidistant from the original two circles of intersection. (This last union $\bigcup \textbf{T}[k,1]$ then has 2k+2 great circles of intersection.) Since the surfaces of [11] are constructed by gluing, all have high genus.

In the present article we adapt the Plateau tiling method used in [20] and, for the odd surfaces, in [4] to construct further desingularizations of $\bigcup \textbf{T}[2,1]$, a union of three Clifford tori intersecting pairwise orthogonally along six great circles. We also adapt arguments from [9] and [10], there used to establish uniqueness and a certain graphicality of the Plateau solutions generating the Lawson surfaces (which can be regarded as desingularizations of intersecting spheres), in order to establish analogous results, namely Theorem 3.25, for solutions to Plateau problems generating desingularizations of intersecting Clifford tori. As one application of Theorem 3.25 we obtain a characterization of the new desingularizations of $\bigcup \textbf{T}[2,1]$ which in particular suffices to confirm that they agree, when of sufficiently high genus, with the members of a certain subfamily of the surfaces we constructed in [11].

### Theorem 1.1

For each integer m≥0 there exists in $\mathbb {S}^3$ a unique closed embedded minimal surface $M_m$ whose intersection with the interior of the prism $\Omega _{00}$ (defined below 4.22 and described in 4.21) is nonempty and satisfies

$$\begin{aligned} \partial (M_m \cap \Omega _{00}) = \Omega _{00} \cap \bigcup (\mathbf {\Gamma }\cup \textbf{L}^{4+8m}) \end{aligned}$$

(where $\mathbf {\Gamma }$ and $\textbf{L}^{4+8m}$ are collections of great circles specified in 4.7-4.8 and 4.17). Furthermore, $M_m$ has genus 25+48m, contains every great circle in $\mathbf {\Gamma }\cup \textbf{L}^{4+8m}$, and is invariant under the group $\mathscr {G}^{4+8m}$ (defined in 4.23 and described in 4.38). Finally, in view of the uniqueness assertion, for all sufficiently large *m* the surface $M_m$ is congruent to the surface in [11] obtained (as in [11, Theorem 7.1]) from the initial surface N(2,2m+1,1,1,1,0,1) (as defined in [11, Definition 4.13]).

Section 4 is devoted to the proof of Theorem 1.1.

As another application of Theorem 3.25, we obtain also a characterization of the odd surfaces of Choe and Soret, which in particular confirms that they agree, when of sufficiently high genus, with the members of a certain subfamily we constructed in [11].

### Theorem 1.2

For all integers k≥2 and m≥1 there is in $\mathbb {S}^3$ a unique closed embedded minimal surface whose intersection with the interior of $\Omega _{\frac{\pi }{4},\frac{\pi }{k},\frac{\pi }{2km}}$ (as defined in 3.7 and described in 3.8) is nonempty and has boundary the hexagon consisting of all the geodesic edges of $\Omega _{\frac{\pi }{4},\frac{\pi }{k},\frac{\pi }{2km}}$ except the one along $C_{(1)}$; this surface is congruent to $T^o_{k,2km}$ (as constructed in [4, Theorem 1]). In particular for all *m* sufficiently large $T^o_{k,2km}$ is congruent to the surface in [11] obtained (as in [11, Theorem 7.1]) from the initial surface *M*(*k*, 2*m*, 1, 1, 0) (as defined in [11, Definition 4.13]).

The proof of Theorem 1.2 is given at the end of Sect. 3.

## Notation and conventions

Given $X \subseteq \mathbb {S}^3$, we write $d^{\mathbb {S}^3}_X$ for the function assigning to each point in $\mathbb {S}^3$ its distance to *X*. Given a great circle *C*, we write $C^\perp$ for the unique great circle on which $d^{\mathbb {S}^3}_C$ is constantly π/2. Given $t \in \mathbb {R}$ together with a great circle *C* and an orientation on $C^\perp$, we write $\textsf{R}_C^t$ for the isometry of $\mathbb {S}^3$ fixing *C* identically and rotating $C^\perp$ along itself through angle *t*; we write ${\underline{\textsf{R}}}_C:=\textsf{R}_C^\pi$ for reflection through *C* (well-defined even in the absence of an orientation on $C^\perp$). If $\{\textsf{T}_\alpha \}_{\alpha \in A}$ is a collection of isometries of $\mathbb {S}^3$ (elements of *O*(4)), we write $\langle \textsf{T}_\alpha \rangle _{\alpha \in A}$ for the subgroup of *O*(4) they generate.

We will make the identifications

2.1 $$\begin{aligned} \begin{aligned} \mathbb {S}^3 := \{ (w_1,w_2) \in \mathbb {C}^2 \; : \; \left\lvert w_1\right\rvert ^2+\left\lvert w_2\right\rvert ^2 = 1 \}, \\ \mathbb {S}^2(1/2) := \left\{ (x,y,z) \in \mathbb {R}^3 \; : \; x^2+y^2+z^2=1/4 \right\} , \end{aligned} \end{aligned}$$

as sets, and we define the Hopf projection

2.2 $$\begin{aligned} \begin{aligned} \Pi _{\textrm{Hopf}}: \mathbb {S}^3 \rightarrow \mathbb {S}^2(1/2) \\ (w_1,w_2) \mapsto \left( \operatorname {Re}w_1\overline{w_2}, ~ \operatorname {Im}w_1\overline{w_2}, ~ \frac{\left\lvert w_1\right\rvert ^2-\left\lvert w_2\right\rvert ^2}{2} \right) , \end{aligned} \end{aligned}$$

a Riemannian submersion, with the usual round metrics on the domain and target.

Throughout the article we distinguish the great circle

2.3 $$\begin{aligned} C_{(1)} := \{(e^{{\textrm{i}}s},0) \in \mathbb {S}^3 \; : \; s \in \mathbb {R}\}, \end{aligned}$$

so that

2.4 $$\begin{aligned} C_{(1)}^\perp := \{(0,e^{{\textrm{i}}t}) \in \mathbb {S}^3 \; : \; t \in \mathbb {R}\}, \end{aligned}$$

and we orient $C_{(1)}$ and $C_{(1)}^\perp$ so that

2.5 $$\begin{aligned} \textsf{R}_C^s(w_1,w_2) = (w_1, e^{{\textrm{i}}s}w_2), \quad \textsf{R}_{C^\perp }^t(w_1,w_2) = (e^{{\textrm{i}}t}w_1,w_2). \end{aligned}$$

The fibers of $\Pi _{\textrm{Hopf}}$ are then the orbits of $\{\textsf{R}_{C_{(1)}}^t\textsf{R}_{C_{(1)}^\perp }^t\}_{t \in \mathbb {R}}$, and, orienting each orbit in the direction of increasing *t*, we have

$$\begin{aligned} \textsf{R}_C^t\textsf{R}_{C^\perp }^t = \textsf{R}_{C_{(1)}}^t\textsf{R}_{C_{(1)}^\perp }^t \end{aligned}$$

for any orbit *C* and any $t \in \mathbb {R}$.

We denote the sets of unitary and antiunitary transformations on $\mathbb {C}^2$ by, respectively,

2.6 $$\begin{aligned} \begin{aligned} U(2)&:= \{ \textsf{T}: \mathbb {C}^2 \rightarrow \mathbb {C}^2 \; : \; \forall x,y \in \mathbb {C}^2 \;\; \langle \textsf{T}x, \textsf{T}y \rangle _{\mathbb {C}^2} = \langle x,y \rangle _{\mathbb {C}^2} \}, \\ \overline{U}(2)&:= \{ \textsf{T}: \mathbb {C}^2 \rightarrow \mathbb {C}^2 \; : \; \forall x,y \in \mathbb {C}^2 \;\; \langle \textsf{T}x, \textsf{T}y \rangle _{\mathbb {C}^2} = \overline{\langle x,y \rangle }_{\mathbb {C}^2} \} \end{aligned} \end{aligned}$$

(where $\langle \cdot ,\cdot \rangle _{\mathbb {C}^2}$ is the standard (Hermitian) inner product on $\mathbb {C}^2$) and we identify them as subsets of *O*(4) in the obvious way. (Then *U*(2) and $U(2) \cup \overline{U}(2)$ are in fact subgroups of *O*(4).) We observe that $\textsf{R}\in O(4)$ preserves the collection of fibers of $\Pi _{\textrm{Hopf}}$ if and only if it is unitary or antiunitary, and we define the map

2.7 $$\begin{aligned} \widehat{\Pi }_{\textrm{Hopf}}: U(2) \cup \overline{U}(2) \rightarrow O(3) \end{aligned}$$

(the target being the group of orthogonal transformations on $\mathbb {R}^3$) which takes any given $\textsf{R}\in U(2) \cup \overline{U}(2)$ to the unique element $\widehat{\Pi }_{\textrm{Hopf}}\textsf{R}$ of *O*(3) satisfying

2.8 $$\begin{aligned} \Pi _{\textrm{Hopf}}\circ \textsf{R}= (\widehat{\Pi }_{\textrm{Hopf}}\textsf{R}) \circ \Pi _{\textrm{Hopf}}. \end{aligned}$$

Then $\widehat{\Pi }_{\textrm{Hopf}}$ is a homomorphism. Note that *U*(2) preserves the orientations of the fibers of $\Pi _{\textrm{Hopf}}$, while $\overline{U}(2)$ reverses them, and that $\widehat{\Pi }_{\textrm{Hopf}}(U(2))=SO(3)$ (the group of orientation-preserving orthogonal transformations on $\mathbb {R}^3$).

## Uniqueness and graphicality of solutions to the Plateau problems

We start with some definitions. First, we set

3.1 $$\begin{aligned} C^0_0 := \{(\cos r, \sin r) \; : \; r \in \mathbb {R}\}, \end{aligned}$$

the great circle orthogonally intersecting $C_{(1)}$ at (±1,0) and $C_{(1)}^\perp$ at (0,±1), and we set

3.2 $$\begin{aligned} C^{\pi /2}_{\pi /2} := (C^0_0)^\perp = \{(i \cos r, i \sin r) \; : \; r \in \mathbb {R}\}. \end{aligned}$$

Next, for each $z \in \mathbb {R}$ we define

3.3 $$\begin{aligned} \Sigma _z := \{ (e^{{\textrm{i}}z}\cos r, e^{{\textrm{i}}\theta } \sin r) \in \mathbb {S}^3 \; : \; r,\theta \in \mathbb {R}\}, \end{aligned}$$

the great sphere containing $C_{(1)}^\perp$ and intersecting $C_{(1)}$ orthogonally at $(\pm e^{{\textrm{i}}z},0)$, and for each $\theta \in \mathbb {R}$ we define also

3.4 $$\begin{aligned} \Sigma ^\theta := \{ (e^{{\textrm{i}}z}\cos r, e^{{\textrm{i}}\theta } \sin r) \in \mathbb {S}^3 \; : \; r,z \in \mathbb {R}\}, \end{aligned}$$

the great sphere containing $C_{(1)}$ and intersecting $C_{(1)}^\perp$ orthogonally at $(0,\pm e^{{\textrm{i}}\theta })$, and

3.5 $$\begin{aligned} \mathbb {T}^\theta := \left\{ e^{{\textrm{i}}z}(\cos r, e^{{\textrm{i}}\theta } \sin r) \in \mathbb {S}^3 \; : \; r,z \in \mathbb {R}\right\} , \end{aligned}$$

a Clifford torus (one of two) containing $C_{(1)} \cup \textsf{R}_{C_{(1)}}^\theta C^0_0$ (and $C_{(1)}^\perp \cup \textsf{R}_{C_{(1)}}^\theta C^{\pi /2}_{\pi /2}$). Given $r \in (0,\pi /2)$, we define

3.6 $$\begin{aligned} T^r := \{d^{\mathbb {S}^3}_{C_{(1)}}=r\} = \{ e^{iz}(\cos r, e^{i\theta }\sin r) \; : \; \theta ,z \in \mathbb {R}\}, \end{aligned}$$

a constant-mean-curvature torus. Finally, given $S \in (0,\pi /4]$, $\Theta \in (0,\pi /2]$, and $Z \in (0,\pi /4]$, we define

3.7 $$\begin{aligned} \begin{array}{c} \Omega _{S,\Theta ,Z}:= \bigg \{ e^{{\textrm{i}}z}(\cos r, e^{{\textrm{i}}\theta } \sin r) \in \mathbb {S}^3 \\ \;: \; r \in [0,S] \text{ and } \theta \in \left[ -\frac{\Theta }{2},\frac{\Theta }{2} \right] \text{ and } z \in \left[ -\frac{Z}{2},\frac{Z}{2} \right] \bigg \}, \end{array}\end{aligned}$$

a sphericotoral pentahedron, or a prism with base an isosceles triangle, as described presently in greater detail. As all claims of the below proposition are simple observations, we do not include a proof.

### Proposition 3.8

Let $S \in (0,\pi /4]$, $\Theta \in (0,\pi /2]$, and $Z \in (0,\pi /4]$. Then $\Omega _{S,\Theta ,Z}$ is homeomorphic to a closed 3-ball and has the following properties:

(i) $\partial \Omega _{S,\Theta ,Z} = R_{S,\Theta ,Z}^- \cup R_{S,\Theta ,Z}^+ \cup T_{S,\Theta ,Z}^- \cup T_{S,\Theta ,Z}^+ \cup P_{S,\Theta ,Z}$, where $R_{S,\Theta ,Z}^{\pm }$ are solid rectangles lying on the two Clifford tori $\mathbb {T}^{\pm \Theta /2}$, $T_{S,\Theta ,Z}^{\pm }$ are solid triangles lying on the two great spheres $\Sigma _{\pm Z/2}$, and $P_{S,\Theta ,Z}$ is a solid parallelogram lying on the constant-mean-curvature torus $T^S$;
(ii) $R_{S,\Theta ,Z}^\pm$ has sides $R_{S,\Theta ,Z}^\pm \cap T_{S,\Theta ,Z}^+$, $R_{S,\Theta ,Z}^\pm \cap T_{S,\Theta ,Z}^-$, $R_{S,\Theta ,Z}^\pm \cap C_{(1)} = R_{S,\Theta ,Z}^+ \cap R_{S,\Theta ,Z}^-$, and $R_{S,\Theta ,Z}^\pm \cap P_{S,\Theta ,Z}$, and vertex angles all of measure π/2;
(iii) $T_{S,\Theta ,Z}^\pm$ has sides $T_{S,\Theta ,Z}^\pm \cap R_{S,\Theta ,Z}^+$, $T_{S,\Theta ,Z}^\pm \cap R_{S,\Theta ,Z}^-$, and $T_{S,\Theta ,Z}^\pm \cap P_{S,\Theta ,Z}$, the first two meeting on $C_{(1)}$ at angle Θ and each of the remaining two vertex angles having measure π/2;
(iv) $P_{S,\Theta ,Z}$ has sides $P_{S,\Theta ,Z} \cap T_{S,\Theta ,Z}^+$, $P_{S,\Theta ,Z} \cap T_{S,\Theta ,Z}^-$, $P_{S,\Theta ,Z} \cap R_{S,\Theta ,Z}^+$, and $P_{S,\Theta ,Z} \cap R_{S,\Theta ,Z}^-$, and vertex angles of measure $\pi /2 \pm S$;
(v) $R_{S,\Theta ,Z}^\pm \cap C_{(1)} = R_{S,\Theta ,Z}^+ \cap R_{S,\Theta ,Z}^-$ has constant dihedral angle Θ and is an arc of length *Z* on the great circle $C_{(1)}$;
(vi) Each of the four edges $R_{S,\Theta ,Z}^{{\pm }_1} \cap T_{S,\Theta ,Z}^{{\pm }_2}$ has dihedral angle varying monotonically between π/2 and $\pi /2 \pm S$ and is an arc of length *S* on a great circle orthogonally intersecting $C_{(1)}$;
(vii) Each of the two edges $P_{S,\Theta ,Z} \cap R_{S,\Theta ,Z}^\pm$ has constant dihedral angle π/2 and is an arc of length *Z* on a great circle on $T^S$;
(viii) Each of the two edges $P_{S,\Theta ,Z} \cap T_{S,\Theta ,Z}^\pm$ has constant dihedral angle π/2 and is an arc of length $\Theta \sin S$, simultaneously a circle of latitude on $\Sigma _{\pm Z/2}$ and a circle of principal curvature on $T^S$;
(ix) $$ {\underline{\textsf{R}}}_{C^0_0}\Omega _{S,\Theta ,Z} = \Omega _{S,\Theta ,Z} $$; and
(x) $\Omega _{S,\Theta ,Z}$ is contained in the closure of a single component of $\mathbb {S}^3 {\setminus } ( \Sigma _{\pi /2} \cup \Sigma ^{\pi /2} )$.

Next we consider the one-parameter group $\Bigl \{\textsf{R}_{C^{\pi /2}_{\pi /2}}^t\Bigr \}_{t \in \mathbb {R}}$ of rotations along $C^0_0$, and we define on $\mathbb {S}^3$ the Killing field *K* by

3.9 $$\begin{aligned} K|_{(w_1,w_2)} := \left. \frac{d}{dt}\right| _{t=0} \textsf{R}_{C^{\pi /2}_{\pi /2}}^t (w_1,w_2) = (-\operatorname {Re}w_2, ~ \operatorname {Re}w_1). \end{aligned}$$

Whenever we refer to an orbit of *K*, it will always be oriented in the direction of *K*. We call $p \in \partial \Omega _{S,\Theta ,Z}$ an entry or exit point of $\Omega _{S,\Theta ,Z}$ for ±K (and say that the ±K orbit of *p* enters or exits $\Omega _{S,\Theta ,Z}$ at *p*) if there exists ϵ>0 such that the set $\{\textsf{R}_{C^{\pi /2}_{\pi /2}}^{\pm t} p\}_{0<t<\epsilon }$ is contained in $\Omega _{S,\Theta ,Z}$ or, respectively, its complement.

### Proposition 3.10

Let $$S \in (0,\pi /4]$$, $$\Theta \in (0,\pi /2]$$, and $$Z \in (0,\Theta /2]$$.

(i) The set of entry points of $\Omega _{S,\Theta ,Z}$ for *K* is $(R^-_{S,\Theta ,Z} \cup R^+_{S,\Theta ,Z}) {\setminus } ( T^-_{S,\Theta ,Z} \cup P_{S,\theta ,Z} \cup T^+_{S,\Theta ,Z} )$,
(ii) The set of exit points of $\Omega _{S,\Theta ,Z}$ for *K* is $T^-_{S,\Theta ,Z} \cup P_{S,\theta ,Z} \cup T^+_{S,\Theta ,Z}$,
(iii) The set of entry points of $\Omega _{S,\Theta ,Z}$ for -K is $( T^-_{S,\Theta ,Z} \cup P_{S,\theta ,Z} \cup T^+_{S,\Theta ,Z} ) {\setminus } (R^-_{S,\Theta ,Z} \cup R^+_{S,\Theta ,Z})$,
(iv) The set of exit points of $\Omega _{S,\Theta ,Z}$ for -K is $R^-_{S,\Theta ,Z} \cup R^+_{S,\Theta ,Z}$,
(v) Each orbit of *K* intersects $T^-_{S,\Theta ,Z} \cup P_{S,\Theta ,Z} \cup T^+_{S,\Theta ,Z}$ in at most one point, and
(vi) Each orbit of *K* through $(R^-_{S,\Theta ,Z} \cup R^+_{S,\Theta ,Z}) \cap ( T^-_{S,\Theta ,Z} \cup P_{S,\Theta ,Z} \cup T^+_{S,\Theta ,Z} )$ intersects $\Omega _{S,\Theta ,Z}$ at a single point.

### Proof

We prefer to present the proof in $\mathbb {R}^4$ rather than $\mathbb {C}^2$ (under the identification $(x_1,x_2,x_3,x_4) \mapsto (x_1+ix_2,x_3+ix_4)$), and we choose to omit the parameters S,Θ,Z from the notation for Ω and each face. We parametrize these faces as follows:

3.11 $$\begin{aligned} \begin{aligned} T^{\pm } := \bigg \{ \bigg ( \cos \frac{Z}{2} \cos r, ~ \pm \sin \frac{Z}{2} \sin r, ~ \qquad \qquad \qquad \qquad \qquad \qquad \qquad \qquad \\ \qquad \qquad \qquad \cos \theta \sin r, ~ \sin \theta \sin r \bigg ) \bigg \}_{ r \in [0,S], \; \theta \in \left[ -\frac{\Theta }{2},\frac{\Theta }{2} \right] }, \\ P := \big \{ ( \cos S \cos z, ~ \cos S \sin z, ~ \qquad \qquad \qquad \qquad \qquad \qquad \qquad \qquad \qquad \\ \qquad \qquad \sin S \cos (z+\theta ), ~ \sin S \sin (z+\theta ) ) \big \}_{ \theta \in \left[ -\frac{\Theta }{2}, \frac{\Theta }{2} \right] , \; z \in \left[ -\frac{Z}{2}, \frac{Z}{2} \right] }, \\ R^{\pm } := \bigg \{ \bigg ( \cos r \cos z, ~ \cos r \sin z, ~ \phantom {k} \\ \qquad \qquad \sin r \cos \left( z \pm \frac{\Theta }{2}\right) , ~ \sin r \sin \left( z \pm \frac{\Theta }{2}\right) \bigg ) \bigg \}_{ r \in [0,S], \; z \in \left[ -\frac{Z}{2}, \frac{Z}{2} \right] }. \end{aligned} \end{aligned}$$

On each face we choose the continuous unit normal which agrees, on the interior of the face, with the outward normal to Ω:

3.12 $$\begin{aligned} \begin{aligned} \nu ^{T^\pm } := \left( -\sin \frac{Z}{2}, ~ \pm \cos \frac{Z}{2}, ~ 0, ~ 0 \right) , \\ \nu ^P := ( -\sin S \cos z, ~ -\sin S \sin z, ~ \cos S \cos (z+\theta ), ~ \cos S \sin (z+\theta ) ), \\ \nu ^{R^\pm } := \bigg ( \pm \sin r \sin z, ~ \mp \sin r \cos z, \phantom {k} \\ \phantom {k} \mp \cos r \sin \left( z \pm \frac{\Theta }{2}\right) , ~ \pm \cos r \cos \left( z \pm \frac{\Theta }{2}\right) \bigg ). \end{aligned} \end{aligned}$$

On the other hand, (making implicit use of the above parametrizations)

3.13 $$\begin{aligned} \begin{aligned} K|_{T^\pm } = \left( -\cos \theta \sin r, ~ 0, ~ \cos \frac{Z}{2} \cos r, ~ 0 \right) , \\ K|_P = \left( -\sin S \cos (z+\theta ), ~ 0, ~ \cos S \cos z, ~ 0 \right) , \\ K|_{R^\pm } = \left( -\sin r \cos \left( z \pm \frac{\Theta }{2}\right) , ~ 0, ~ \cos r \cos z, ~ 0 \right) . \end{aligned} \end{aligned}$$

Recalling that $0 \le r \le S \le \pi /4$, $\left\lvert \theta \right\rvert \le \Theta /2 \le \pi /4$, and $\left\lvert z\right\rvert \le Z/2 \le \Theta /4$, we then compute

3.14 $$\begin{aligned} K|_{T^\pm } \cdot \nu ^{T^\pm } = \sin \frac{Z}{2} \sin r \cos \theta \ge 0, \quad \text {with equality iff } r=0 , \end{aligned}$$

3.15 $$\begin{aligned} K|_P \cdot \nu ^P = \cos z \cos (z+\theta ) > 0, \end{aligned}$$

3.16 $$\begin{aligned} \begin{aligned} K|_{R^\pm } \cdot \nu ^{R^\pm }&= - \left( \cos ^2 r \sin \frac{\Theta }{2} \pm \sin z \cos \left( z \pm \frac{\Theta }{2}\right) \right) \\&= -\frac{1}{2} \left( \sin \frac{\Theta }{2} \cos 2r +\sin \left( \frac{\Theta }{2} \pm 2z\right) \right) \\&\le 0, \quad \text {with equality iff } r=S=\frac{\pi }{4} \text { and } z=\frac{\mp Z}{2}=\frac{\mp \Theta }{4} \text  . \end{aligned} \end{aligned}$$

The above inequalities establish items i, ii, iii, and iv except at the two points

3.17 $$\begin{aligned} T^\pm \cap R^+ \cap R^- = (\pm Z/2,0,0,0) \end{aligned}$$

and, in the case that $S=\frac{\pi }{4}$ and $Z=\frac{\Theta }{2}$, at the two additional points

3.18 $$\begin{aligned} R^\pm \cap T^\mp \cap P = \frac{1}{\sqrt{2}} \left( \cos \frac{\Theta }{4}, ~ \mp \sin \frac{\Theta }{4}, ~ \cos \frac{\Theta }{4}, ~ \pm \sin \frac{\Theta }{4} \right) . \end{aligned}$$

The orbit of $T^\pm \cap R^+ \cap R^-$ is, according to the above calculation, tangential there to $\Sigma _{\pm Z/2}$ but is easily seen (since 0<z<π/2) to have, at the same point, vector-valued curvature directed along $C_{(1)}$ away from (1, 0, 0, 0), while $\Sigma _{\pm Z/2}$ is totally geodesic, confirming that $T^\pm \cap R^+ \cap R^-$ is an exit point for both *K* and -K. In fact this completes the proof of i and ii.

In the case that $S=\frac{\pi }{4}$ and $Z=\frac{\Theta }{2}$ the orbit through $R^\pm \cap T^{\mp } \cap P$ is parametrized by

3.19 $$\begin{aligned} \alpha _\pm (t) = \frac{1}{\sqrt{2}} \left( (\cos t-\sin t) \cos \frac{\Theta }{4}, ~ \mp \sin \frac{\Theta }{4}, ~ (\cos t + \sin t)\cos \frac{\Theta }{4}, ~ \pm \sin \frac{\Theta }{4} \right) , \end{aligned}$$

while $\mathbb {T}^{\pm \Theta /2}$ is the intersection with $\mathbb {S}^3$ of the null set of

$$\begin{aligned} f_\pm (x_1,x_2,x_3,x_4) := (x_2x_3 - x_1 x_4) \cos \frac{\Theta }{2} \pm (x_1x_3 + x_2x_4) \sin \frac{\Theta }{2}; \end{aligned}$$

then

3.20 $$\begin{aligned} f_\pm (\alpha _\pm (t)) = \pm \frac{1}{2}\left( \sin \frac{\Theta }{2}\right) \left( \cos ^2 \frac{\Theta }{4} \cos 2t -\cos \frac{\Theta }{2} \cos t -\sin ^2 \frac{\Theta }{4} \right) , \end{aligned}$$

and so

3.21 $$\begin{aligned} \mp 2\left. \frac{d^2}{dt^2}\right| _{t=0}f_\pm (\alpha _\pm (t)) = \left( \sin \frac{\Theta }{2}\right) \left( 1+2\cos ^2 \frac{\Theta }{4} \right) > 0, \end{aligned}$$

but $\pm f_\pm |_\Omega \ge 0$, completing the proof of claims iii and iv.

We turn to claim v. Using the fact that the orbit of $(x_1,x_2,x_3,x_4)$ can be parametrized by

3.22 $$\begin{aligned} \beta (t) := ( x_1 \cos t - x_3 \sin t, ~ x_2, ~ x_1 \sin t + x_3 \cos t, ~ x_4 ), \end{aligned}$$

we observe that if $(x_1,x_2,x_3,x_4) \in \Sigma ^{\pm Z/2}$, then $x_2 = \pm x_1 \tan \frac{Z}{2}$ and $\beta (t) \in \Sigma ^{\pm Z/2}$ when

3.23 $$\begin{aligned} x_1 \cos t - x_3 \sin t = x_1, \end{aligned}$$

which has two solutions (modulo $$2\pi \mathbb {Z}$$) unless $$x_3=0$$, in which case only one solution. The computation 3.14 and the analysis in the paragraph immediately following 3.18 reveal that at t=0 the orbit β(t) not only exits Ω but also enters the component $$\mathbb {S}^3 \setminus \Sigma ^{\pm Z/2}$$ disjoint from Ω. Thus the least strictly positive *t*, at which $$\beta (t) \in T^+ \cup P \cup T^-$$, which we will call $t^{\prime }$, is at least the least positive *t* satisfying 3.22. Since $$x_1,x_3 \ge 0$$ (simply because $$(x_1,x_2,x_3,x_4) \in \Omega $$), this last value is strictly greater than π. On the other hand, the order-4 subgroup $$ \left\langle \textsf{R}_{C^{\pi /2}_{\pi /2}}^{\pi /2} \right\rangle $$ acts simply transitively on the components of $$\mathbb {S}^3 \setminus (\Sigma _{\pi /2} \cup \Sigma ^{\pi /2})$$, a single one of which contains the interior of Ω. It follows that $$t' \ge 3\pi /2$$.

Now suppose that $(x_1,x_2,x_3,x_4) \in P$. Then $x_1^2+x_2^2 = \cos ^2 S$, and $\beta (t) \in T^S$ when

3.24 $$\begin{aligned} (\sin t) \left[ (x_3^2-x_1^2) \sin t - 2x_1x_3 \cos t \right] = 0, \end{aligned}$$

which (under the assumption $(x_1,x_2,x_3,x_4) \in P$) has four solutions (modulo $2\pi \mathbb {Z}$). Since both ${\underline{\textsf{R}}}_{\Sigma _{\pi /2}}$ and ${\underline{\textsf{R}}}_{\Sigma ^{\pi /2}}$ are symmetries of both $T^S$ and $C^0_0$, each component of $\mathbb {S}^3 \setminus (\Sigma _{\pi /2} \cup \Sigma ^{\pi /2})$ contains a solution. On the other hand, 3.15 shows that at t=0 the orbit β exits the component of $\mathbb {S}^3 \setminus T^S$ containing Ω. It follows that the least strictly positive *t* for which $\beta (t) \in T^- \cup P \cup T^+$ is at least 3π/2. Since each nontrivial orbit of *K* has period 2π, the conclusions of this and the preceding paragraph show that intersection of any orbit of *K* with $T^- \cup P \cup T^+$ has cardinality at most one, confirming v.

Finally, let *O* be an orbit through a point $p \in (R^- \cup R^+) \cap (T^- \cup P \cup T^+)$. In particular, by ii, *p* is an exit point for *K*. By v, if $O \cap \Omega \ne \{p\}$, then there is also an entry point for *K* on *O*, and then *p* would also have to an entry point for -K, violating iv and so confirming vi. □

Now we can prove uniqueness and *K* graphicality (as defined in the statement below) of the solution to a certain Plateau problem in $\Omega _{S,\Theta ,Z}$.

### Theorem 3.25

Let $S \in (0,\pi /4]$, $\Theta \in (0,\pi /2]$, and $Z \in (0,\Theta /2]$. Then there is a unique connected minimal surface (with boundary) *D*, an embedded disc, such that $D \subset \Omega _{S,\Theta ,Z}$ and

$$\begin{aligned} \begin{aligned} \partial D = (R^-_{S,\Theta ,Z} \cup R^+_{S,\Theta ,Z}) \cap ( T^-_{S,\Theta ,Z} \cup P_{S,\Theta ,Z} \cup T^+_{S,\Theta ,Z} ), \end{aligned} \end{aligned}$$

the hexagon which is the union of the edges of $\Omega _{S,\Theta ,Z}$ except $R_{S,\Theta ,Z}^- \cap R_{S,\Theta ,Z}^+$, $P_{S,\Theta ,Z} \cap T_{S,\Theta ,Z}^+$, and $P_{S,\Theta ,Z} \cap T_{S,\Theta ,Z}^-$, Furthermore, *D* is graphical with respect to *K* in the sense that no orbit of *K* intersects *D* in more than one point.

### Proof

By [22, Theorem 1] there exists an embedded minimal disc $$D \subset \Omega _{S,\Theta ,Z}$$ with the prescribed boundary. Now suppose *M* is a connected minimal surface in $$\Omega _{S,\Theta ,Z}$$ with $$\partial M = \partial D$$. We will show, by contradiction, that no orbit of *K* intersects *D* and *M* at distinct points. To this end suppose then that such an orbit does exist. This assumption, together with the fact that $$\Omega _{S,\Theta ,Z}$$ lies in the closure of a single component of $$\mathbb {S}^3 \setminus (\Sigma _{\pi /2} \cup \Sigma ^{\pi /2})$$, implies the existence of $$t' \in \mathbb {R}\setminus 2\pi \mathbb {Z}$$ and ϵ>0 such that the intersection $$D \cap \textsf{R}_{C^{\pi /2}_{\pi /2}}^t M$$ is nonempty for $t=t^{\prime }$ but empty either for $$t \in (t',t'+\epsilon )$$ or $$t \in (t'-\epsilon , t')$$. Item vi of 3.10 ensures that the intersection, when $t=t^{\prime }$, cannot include any points in the boundary of either surface (*D* or the rotated *M*). The maximum principle then forces the surfaces to coincide, but since $$t' \not \in 2\pi \mathbb {Z}$$, their boundaries, again by 3.10.vi, are actually disjoint, which contradiction establishes our claim that no orbit of *K* intersects *D* and *M* at distinct points.

The graphicality claim is simply the special case of this last fact when M=D. To prove the uniqueness, note that it follows from the embeddedness of *D*, the fact that $\Omega _{S,\Theta ,Z}$ is a 3-ball, and items i and ii of 3.10 that each orbit of *K* intersecting $\Omega _{S,\Theta ,Z}$ also intersects *D*, and so, if M≠D, there would exist an orbit of *K* intersecting *M* and *D* at distinct points, which contradiction completes the proof. □

### **Proof of Theorem**1.2

Now Theorem 1.2 is obtained as a corollary of Theorem 3.25 as follows. Choe and Soret construct $T^o_{k,2km}$ (in the proof of [4, Theorem 1]) by (i) solving the Plateau problem, for an embedded disc—called *H* in [4]—in a prism—$U_1^1$ in the notation of [4]—congruent to $\Omega _{\pi /4,\pi /k,\pi /2km}$ with prescribed boundary a geodesic hexagon as in Theorem 3.25 (shifted by the congruence) and then (ii) checking that *H* can be extended to a closed embedded surface by applying the group—$G^o$ in [4]—generated by the reflections through the great circles containing the sides of the hexagon. Theorem 3.25 establishes that the solution to the preceding Plateau problem is unique, and so the surface $T^o_{k,2km}$ is uniquely determined by its construction.

Similarly, from the reflection principle it is clear that any closed embedded minimal surface whose intersection with $U_1^1$ is nonempty and has boundary ∂H is uniquely determined by its intersection with $U_1^1$, but Theorem 3.25 implies that this intersection is *H*, and so the surface is $T^o_{k,2km}$.

Finally, to see that $T^o_{k,2km}$ agrees with the minimal surface constructed in [11, Theorem 7.1] from the initial surface *M*(*k*, 2*m*, 1, 1, 0) ([11, Definition 4.13]) under the assumption that *m* is large enough to ensure the existence of the latter surface, it suffices to confirm that, modulo congruence, the intersection of the latter surface with $U_1^1$ is nontrivial and has boundary ∂H. For this of course it is necessary to refer to some of the details of [11], but we explain here the key points to check.

In [11] the initial surface *M*(*k*, 2*m*, 1, 1, 0) is constructed explicitly and then graphically perturbed to minimality by solving the corresponding nonlinear partial differential equation, first by constructing a suitable right-inverse to the linearized operator and then by appealing to the Schauder fixed point theorem to solve the nonlinear problem. The explicit construction (see [11, 4.14] and the supporting definitions) of the initial surface *M*(*k*, 2*m*, 1, 1, 0) makes it clear that *M*(*k*, 2*m*, 1, 1, 0) (modulo congruence) not only has the desired properties that its intersection with $U_1^1$ is nontrivial and has boundary ∂H but moreover is invariant under $G^o$.

In [11] we enforce a proper subgroup of $G^o$, but it is easy to see, given the $G^o$ invariance of this initial surface, that in this case the larger group $G^o$ could be enforced. (In fact the right-inverse to the linearized problem constructed in [11, Section 6] respects $G^o$, without modification.) Although in [11] we did not prove uniqueness of the minimal perturbations of the initial surfaces considered there, one could in this case simply appeal to the inverse function theorem in place of the Schauder fixed point theorem, and thereby establish uniqueness, assuming *m* sufficiently large. Thus we conclude that the minimal surface obtained from *M*(*k*, 2*m*, 1, 1, 0) is invariant under $G^o$, but since it is also a small perturbation of a surface whose intersection with $U_1^1$ is nonempty and has boundary *H*, its own intersection with $U_1^1$ will have these same properties, completing the proof.□

## Desingularizations of three Clifford tori intersecting pairwise orthogonally

### **Initial configuration**

**Fig. 1:**
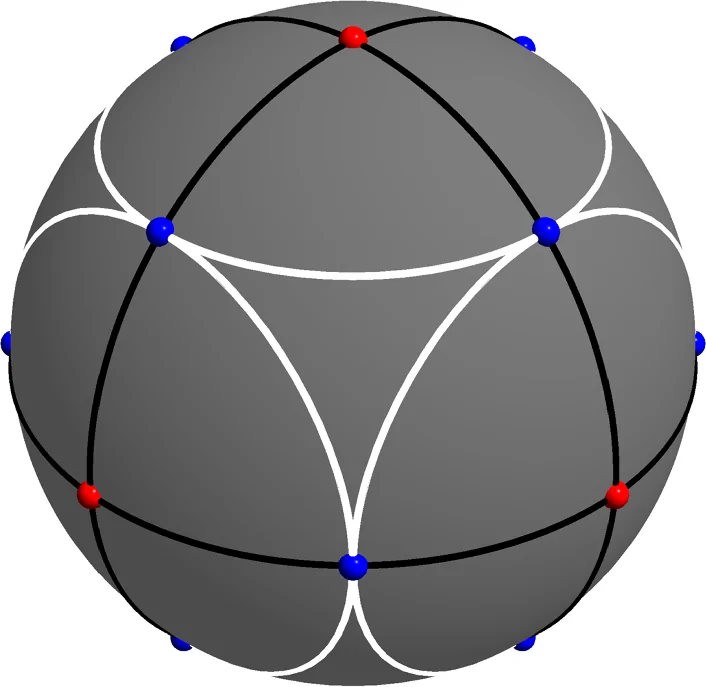
The images under $\Pi _{\textrm{Hopf}}$ of $\{\mathbb {T}_i\}_{i=1}^3$ (in black), $\textbf{C}$ (red). $\{\partial T_C\}_{C \in \textbf{C}}$ (white), and $\mathbf {\Gamma }$ (blue)

Fix a Clifford torus $\mathbb {T}_2$ containing $C_{(1)}$. There is a unique Clifford torus $\mathbb {T}_3 = \textsf{R}_{C_{(1)}}^{\pi /2}\mathbb {T}_2 = \textsf{R}_{C_{(1)}}^{-\pi /2}\mathbb {T}_2$ intersecting $\mathbb {T}_2$ orthogonally along $C_{(1)}$. Set $\mathbb {T}_1:=\{d^{\mathbb {S}^3}_{C_{(1)}}=\pi /4\}$, another Clifford torus (see Fig. 1). More concretely, we will take

4.1 $$\begin{aligned} \mathbb {T}_2 := \{(e^{{\textrm{i}}s} \cos r, e^{{\textrm{i}}s} \sin r)\}_{r,s \in \mathbb {R}} = \Pi _{\textrm{Hopf}}^{-1}(\{y=0\}), \end{aligned}$$

and so

4.2 $$\begin{aligned} \begin{aligned} \mathbb {T}_1 = \{2^{-1/2}(e^{{\textrm{i}}s}, e^{{\textrm{i}}t})\}_{s,t \in \mathbb {R}} = \Pi _{\textrm{Hopf}}^{-1}(\{z=0\}), \\ \mathbb {T}_3 = \{e^{{\textrm{i}}s}(\cos r, {\textrm{i}}\sin r)\}_{r,s \in \mathbb {R}} = \Pi _{\textrm{Hopf}}^{-1}(\{x=0\}). \end{aligned} \end{aligned}$$

The following claims are immediate consequences of the foregoing definitions.

We have the following (see Fig. 1).

#### Proposition 4.3

  

(i) The set $\bigcup _{i \ne j} (\mathbb {T}_i \cap \mathbb {T}_j)$ consists of six pairwise disjoint great circles, the collection of which we call $\textbf{C}$.
(ii) Each of the three $\mathbb {T}_i$ coincides with $\{d^{\mathbb {S}^3}_C=\pi /4\}=\{d^{\mathbb {S}^3}_{C^\perp }=\pi /4\}$ for precisely two choices of $C \in \textbf{C}$, along each of which the other two tori intersect orthogonally.
(iii) For any $$C,C' \in \textbf{C}$$ the function $$d^{\mathbb {S}^3}_C$$ is constant on $C^{\prime }$.
(iv) Each $C \in \textbf{C}$ has four nearest neighbors (excluding *C* itself) in $\textbf{C}$, each at distance π/4, namely $(\bigcup \textbf{C}) \setminus (C \cup C^\perp )$.

Namely, we have

4.4 $$\begin{aligned} \begin{aligned} \textbf{C}= \{C_{(i)}\}_{i=1}^3 \cup \{C_{(i)}^\perp \}_{i=1}^3, \\ C_{(2)} := \left\{ \frac{e^{{\textrm{i}}s}}{\sqrt{2}} (1,{\textrm{i}}) \right\} _{s \in \mathbb {R}} = \Pi _{\textrm{Hopf}}^{-1}(\{(0,-\tfrac{1}{2},0)\}), \\ C_{(2)}^\perp = \left\{ \frac{e^{{\textrm{i}}s}}{\sqrt{2}} (1,-{\textrm{i}}) \right\} _{s \in \mathbb {R}} = \Pi _{\textrm{Hopf}}^{-1}(\{(0,\tfrac{1}{2},0)\}), \\ C_{(3)} := \left\{ \frac{e^{{\textrm{i}}s}}{\sqrt{2}} (1,1) \right\} _{s \in \mathbb {R}} = \Pi _{\textrm{Hopf}}^{-1}(\{(\tfrac{1}{2},0,0)\}), \\ C_{(3)}^\perp = \left\{ \frac{e^{{\textrm{i}}s}}{\sqrt{2}} (1,-1) \right\} _{s \in \mathbb {R}} = \Pi _{\textrm{Hopf}}^{-1}(\{(-\tfrac{1}{2},0,0)\}). \end{aligned} \end{aligned}$$

### **Packing**

For each $C \in \textbf{C}$ define the closed solid torus

4.5 $$\begin{aligned} T_C := \{d^{\mathbb {S}^3}_C \le \pi /8\} \quad (C \in \textbf{C}). \end{aligned}$$

We have the following (see Fig. 1).

#### Proposition 4.6

  

(i) For any $C \ne C' \in \textbf{C}$ the intersection $T_C \cap T_{C'}$ is empty if $C'=C^\perp$ and otherwise consists of a single great circle lying on $\partial T_C \cap \partial T_{C'}$. In particular $\{T_C : \; C \in \textbf{C}\}$ consists of six solid tori, whose interiors are pairwise disjoint.
(ii) We have the equality
  $$\begin{aligned} \bigcup _{C \ne C' \in \textbf{C}} (T_C \cap T_{C'}) = \bigcup _{i=1}^3 \bigcup _{C \in \textbf{C}} (\mathbb {T}_i \cap \partial T_C), \end{aligned}$$
  and this set consists of twelve great circles, the collection of which we call $\mathbf {\Gamma }$.

#### Proof

Suppose $$C \ne C' \in \textbf{C}$$. and $$p \in T_C \cap T_{C'}$$. Then the distance between *C* and $C^{\prime }$ is at most π/4, so in fact $C^{\prime }$ must be one of the four nearest neighbors of *C* in $$\textbf{C}$$ and therefore $$d^{\mathbb {S}^3}_C|_{C'}=\pi /4$$. Moreover, *p* must lie on a great circle intersecting *C* and $C^{\prime }$ orthogonally, and so *p* lies on the unique torus $$\mathbb {T}\in \{\mathbb {T}_1,\mathbb {T}_2,\mathbb {T}_3\}$$ containing $C\cup C^{\prime }$. We have $$ \mathbb {T}\cap \{d^{\mathbb {S}^3}_C=\pi /8\} = \mathbb {T}\cap \{d^{\mathbb {S}^3}_{C'}=\pi /8\} $$, and this set is a great circle. Finally, each $$\mathbb {T}_i$$ is disjoint from two of the six tori in $$\{T_{C''}\}_{C'' \in \textbf{C}}$$ and intersects the boundaries of the remaining four in a total of four great circles, while the $$\mathbb {T}_i$$ themselves intersect (in pairs) only along the circles in $$\textbf{C}$$. □

Explicitly,

4.7 $$\begin{aligned} \begin{aligned} \mathbf {\Gamma }= \{ \Gamma _{12}, \Gamma _{13}, \Gamma _{12^\perp }, \Gamma _{13^\perp }, \Gamma _{1^\perp 2}, \Gamma _{1^\perp 3}, \Gamma _{1^\perp 2^\perp }, \Gamma _{1^\perp 3\perp }, \Gamma _{23}, \Gamma _{23^\perp }, \Gamma _{2^\perp 3}, \Gamma _{2^\perp 3^\perp } \}, \\ \Gamma _{12} := \left\{ e^{{\textrm{i}}s} \left( \cos \frac{\pi }{8}, {\textrm{i}}\sin \frac{\pi }{8} \right) \right\} _{s \in \mathbb {R}} = \Pi _{\textrm{Hopf}}^{-1} \left( \left\{ \frac{\sqrt{2}}{4}(0,-1,1) \right\} \right) , \\ \Gamma _{1^\perp 2^\perp } := \left\{ e^{{\textrm{i}}s} \left( \sin \frac{\pi }{8}, -{\textrm{i}}\cos \frac{\pi }{8} \right) \right\} _{s \in \mathbb {R}} = \Pi _{\textrm{Hopf}}^{-1} \left( \left\{ \frac{\sqrt{2}}{4}(0,1,-1) \right\} \right) = \Gamma _{12}^\perp , \\ \Gamma _{13} := \left\{ e^{{\textrm{i}}s} \left( \cos \frac{\pi }{8}, \sin \frac{\pi }{8} \right) \right\} _{s \in \mathbb {R}} = \Pi _{\textrm{Hopf}}^{-1} \left( \left\{ \frac{\sqrt{2}}{4}(1,0,1) \right\} \right) , \\ \Gamma _{1^\perp 3^\perp } := \left\{ e^{{\textrm{i}}s} \left( \sin \frac{\pi }{8}, -\cos \frac{\pi }{8} \right) \right\} _{s \in \mathbb {R}} = \Pi _{\textrm{Hopf}}^{-1} \left( \left\{ \frac{\sqrt{2}}{4}(-1,0,-1) \right\} \right) = \Gamma _{13}^\perp , \\ \Gamma _{12^\perp } := \left\{ e^{{\textrm{i}}s} \left( \cos \frac{\pi }{8}, -{\textrm{i}}\sin \frac{\pi }{8} \right) \right\} _{s \in \mathbb {R}} = \Pi _{\textrm{Hopf}}^{-1} \left( \left\{ \frac{\sqrt{2}}{4}(0,1,1) \right\} \right) , \end{aligned} \end{aligned}$$

4.8 $$\begin{aligned} \begin{aligned} \Gamma _{1^\perp 2} := \left\{ e^{{\textrm{i}}s} \left( \sin \frac{\pi }{8}, {\textrm{i}}\cos \frac{\pi }{8} \right) \right\} _{s \in \mathbb {R}} = \Pi _{\textrm{Hopf}}^{-1} \left( \left\{ \frac{\sqrt{2}}{4}(0,-1,-1) \right\} \right) = \Gamma _{12^\perp }^\perp , \\ \Gamma _{13^\perp } := \left\{ e^{{\textrm{i}}s} \left( \cos \frac{\pi }{8}, -\sin \frac{\pi }{8} \right) \right\} _{s \in \mathbb {R}} = \Pi _{\textrm{Hopf}}^{-1} \left( \left\{ \frac{\sqrt{2}}{4}(-1,0,1) \right\} \right) , \\ \Gamma _{1^\perp 3} := \left\{ e^{{\textrm{i}}s} \left( \sin \frac{\pi }{8}, \cos \frac{\pi }{8} \right) \right\} _{s \in \mathbb {R}} = \Pi _{\textrm{Hopf}}^{-1} \left( \left\{ \frac{\sqrt{2}}{4}(1,0,-1) \right\} \right) = \Gamma _{13^\perp }^\perp , \\ \Gamma _{23} := \left\{ \frac{e^{{\textrm{i}}s}}{\sqrt{2}} (1,e^{{\textrm{i}}\pi /4}) \right\} _{s \in \mathbb {R}} = \Pi _{\textrm{Hopf}}^{-1} \left( \left\{ \frac{\sqrt{2}}{4}(1,-1,0) \right\} \right) , \\ \Gamma _{2^\perp 3^\perp } := \left\{ \frac{e^{{\textrm{i}}s}}{\sqrt{2}} (1,e^{5{\textrm{i}}\pi /4}) \right\} _{s \in \mathbb {R}} = \Pi _{\textrm{Hopf}}^{-1} \left( \left\{ \frac{\sqrt{2}}{4}(-1,1,0) \right\} \right) = \Gamma _{23}^\perp , \\ \Gamma _{2^\perp 3} := \left\{ \frac{e^{{\textrm{i}}s}}{\sqrt{2}} (1,e^{-{\textrm{i}}\pi /4}) \right\} _{s \in \mathbb {R}} = \Pi _{\textrm{Hopf}}^{-1} \left( \left\{ \frac{\sqrt{2}}{4}(1,1,0) \right\} \right) , \\ \Gamma _{23^\perp } := \left\{ \frac{e^{{\textrm{i}}s}}{\sqrt{2}} (1,e^{3{\textrm{i}}\pi /4}) \right\} _{s \in \mathbb {R}} = \Pi _{\textrm{Hopf}}^{-1} \left( \left\{ \frac{\sqrt{2}}{4}(-1,-1,0) \right\} \right) = \Gamma _{2^\perp 3}^\perp . \end{aligned} \end{aligned}$$

### **Scaffolding**

Now let N≥1 be a given integer. We fix a set $\textbf{p}^N_{C_{(1)}}$ of 2*N* equally spaced points on $C_{(1)}$. (In particular $\textbf{p}^N_{C_{(1)}}$ is invariant under antipodal reflection.) We take

4.9 $$\begin{aligned} \textbf{p}^N_{C_{(1)}} := \{(e^{j\pi {\textrm{i}}/N},0)\}_{j=1}^{2N}. \end{aligned}$$

Then there are unique collections $\textbf{L}^N_{\mathbb {T}_2}$ and $\textbf{L}^N_{\mathbb {T}_3}$ of great circles on $\mathbb {T}_2$ and $\mathbb {T}_3$ respectively such that $\bigcup _{L \in \textbf{L}^N_{\mathbb {T}_i}} (L \cap C_{(1)}) = \textbf{p}^N_{C_{(1)}}$ for each $i \in \{2,3\}$. Each of these collections contains *N* great circles, and each of these great circles intersects $C_{(1)}$ orthogonally and also $C_{(1)}^\perp$ orthogonally at two points. We have

4.10 $$\begin{aligned} \textbf{L}^N_{\mathbb {T}_2} = \{ \{ e^{j\pi {\textrm{i}}/N}(\cos r, \sin r) \}_{r \in \mathbb {R}} \}_{j=1}^N, \quad \textbf{L}^N_{\mathbb {T}_3} = \{ \{ e^{j\pi {\textrm{i}}/N}(\cos r, {\textrm{i}}\sin r) \}_{r \in \mathbb {R}} \}_{j=1}^N. \end{aligned}$$

#### Proposition 4.11

If *N* is even, then $\bigcup _{L \in \textbf{L}^N_{\mathbb {T}_2}} (L \cap C_{(1)}^\perp ) = \bigcup _{L \in \textbf{L}^N_{\mathbb {T}_3}} (L \cap C_{(1)}^\perp )$ and consists of 2*N* equally spaced points, which we call $\textbf{p}^N_{C_{(1)}^\perp }$.

#### Proof

Two circles meeting one another and $C_{(1)}$ orthogonally at two points meet $C_{(1)}^\perp$ orthogonally and (collectively) at four equally spaced points. □

We henceforth assume *N* even. We have

4.12 $$\begin{aligned} \textbf{p}^N_{C_{(1)}^\perp } = \{(0,e^{j\pi {\textrm{i}}/N})\}_{j=1}^{2N}. \end{aligned}$$

For each $i \in \{2,3\}$ each $L \in \textbf{L}^N_{\mathbb {T}_i}$ also orthogonally intersects each of the two circles in $\textbf{C}$ along which $\mathbb {T}_i$ and $\mathbb {T}_1$ intersect. For each $C \in \textbf{C}$ we set

4.13 $$\begin{aligned} \textbf{p}^N_C := \bigcup _{i=2}^3 \bigcup _{L \in \textbf{L}^N_{\mathbb {T}_i}} (L \cap C), \end{aligned}$$

a collection of 2*N* equally spaced points, consistent with the pre-existing definitions in case $C \in \{C_{(1)},C_{(1)}^\perp \}$. We further define

4.14 $$\begin{aligned} \textbf{p}^N := \bigcup _{C \in \textbf{C}} \textbf{p}^N_C. \end{aligned}$$

Then

4.15 $$\begin{aligned} \begin{aligned} \textbf{p}^N_{C_{(2)}} = \{ e^{j \pi {\textrm{i}}/ N} (1,{\textrm{i}}) / \sqrt{2} \}_{j=1}^{2N}, \quad \textbf{p}^N_{C_{(2)}^\perp } = \{ e^{j \pi {\textrm{i}}/ N} (1,-{\textrm{i}}) / \sqrt{2} \}_{j=1}^{2N}, \\ \textbf{p}^N_{C_{(3)}} = \{ e^{j \pi {\textrm{i}}/ N} (1,1) / \sqrt{2} \}_{j=1}^{2N}, \quad \textbf{p}^N_{C_{(3)}^\perp } = \{ e^{j \pi {\textrm{i}}/ N} (1,-1) / \sqrt{2} \}_{j=1}^{2N}. \end{aligned} \end{aligned}$$

#### Proposition 4.16

Let *N* be divisible by 4. Then for any of the four $C \in \textbf{C}$ on $\mathbb {T}_1$ the *N* great circles on $\mathbb {T}_1$ orthogonally intersecting *C* (collectively) on $\textbf{p}^N_C$ intersect (collectively) each circle $C' \in \textbf{C}$ on $\mathbb {T}_1$ in precisely $\textbf{p}^N_{C'}$. Thus the collection of these *N* great circles, which we call $\textbf{L}^N_{\mathbb {T}_1}$, is independent of *C*.

#### Proof

Suppose $$C \in \textbf{C}$$ and $$C \subset \mathbb {T}_1$$ and suppose $$L \subset \mathbb {T}_1$$ is a great circle intersecting *C* orthogonally at $$\pm p \in \textbf{p}^N_C$$. Since *N* is even, $$L \cap C^\perp \subset \textbf{p}^N_{C^\perp }$$ (as in 4.11), so suppose $C^{\prime }$ is one of the two circles in $$\textbf{C}$$ on $$\mathbb {T}_1$$ and at distance π/4 from *C*. We may assume $$C'=\textsf{R}_{C_{(1)}}^{-\pi /2}C$$. Then *L* intersects $C^{\prime }$ at $$p' = \textsf{R}_{C_{(1)}}^{-\pi /4}\textsf{R}_{C_{(1)}^\perp }^{\pi /4}p$$ and $-p^{\prime }$. Since $$p \in \textbf{p}^N_C$$ and Nmod4=0, we also have $$q:=\textsf{R}_{C_{(1)}}^{\pi /4}\textsf{R}_{C_{(1)}^\perp }^{\pi /4}p \in C$$, but then by definition of $$\textbf{p}^N_C$$ and $$\textbf{p}^N_{C'}$$ the point $$p'=\textsf{R}_{C_{(1)}}^{-\pi /2}q$$ indeed belongs to $$\textbf{p}^N_{C'}$$. □

We henceforth assume *N* divisible by 4. Set

4.17 $$\begin{aligned} \textbf{L}^N := \bigcup _{i=1}^3 \textbf{L}^N_{\mathbb {T}_i}, \end{aligned}$$

a collection of 3*N* great circles. We have

4.18 $$\begin{aligned} \bigcup _{L \ne L' \in \textbf{L}^N} (L \cap L') = \bigcup _{L \in \textbf{L}^N} \bigcup _{C \in \textbf{C}} L \cap C = \textbf{p}^N. \end{aligned}$$

Concretely,

4.19 $$\begin{aligned} \textbf{L}^N_{\mathbb {T}_1} = \left\{ \left\{ \frac{e^{j\pi {\textrm{i}}/N}}{\sqrt{2}} (e^{{\textrm{i}}s}, e^{-{\textrm{i}}s}) \right\} _{s \in \mathbb {R}} \right\} _{j=1}^N. \end{aligned}$$

### **Tessellations**

For each $C \in \textbf{C}$ let $\mathbf {\Sigma }^N_C$ be the collection of the 2*N* connected components of the intersections with the solid torus $T_C$ of the *N* great spheres (collectively) intersecting *C* orthogonally on the set $\textbf{p}^N_C$; each element of $\mathbf {\Sigma }^N$ is thus a closed spherical cap of radius π/8. Then $T_C \setminus \bigcup \mathbf {\Sigma }^N_C$ has 2*N* congruent components, and $T_C \setminus (\bigcup \mathbf {\Sigma }^N_C \cup \bigcup _{i=1}^3 \mathbb {T}_i)$ has 8*N* congruent components, the collection of whose closures we call $\mathbf {\Omega }^N_C$. We also define

4.20 $$\begin{aligned} \mathbf {\Sigma }^N := \bigcup _{C \in \textbf{C}} \mathbf {\Sigma }^N_C, \quad \mathbf {\Omega }^N := \bigcup _{C \in \textbf{C}} \mathbf {\Omega }^N_C. \end{aligned}$$

The below description of the 48*N* congruent members of $\mathbf {\Omega }^N$ follows immediately from the preceding definitions.

#### Proposition 4.21

Assume $N \in 4\mathbb {Z}$ positive. Let $C \in \textbf{C}$ and $\Omega \in \mathbf {\Omega }^N_C$. Then

(i) Ω is congruent to $\Omega _{\frac{\pi }{8},\frac{\pi }{2},\frac{\pi }{N}}$ (as defined in 3.7);
(ii) The two rectangular faces $R_\Omega ^\pm$ lie on two (minimal) tori in $\{\mathbb {T}_i\}_{i=1}^3$, the two triangular faces $T_\Omega ^\pm$ lie on two members of $\mathbf {\Sigma }^N_C$, and the parallelogram face $P_\Omega$ lies on the torus (of nonzero constant mean curvature) $\partial T_C$;
(iii) Each of the four edges $R_\Omega ^{{\pm }_1} \cap T_\Omega ^{{\pm }_2}$ is an arc of length π/8 on a great circle in $\textbf{L}^N$;
(iv) Each of the two edges $P_\Omega \cap R_\Omega ^\pm$ has constant dihedral angle π/2 and is an arc of length π/N on a great circle in $\mathbf {\Gamma }$;
(v) For any given edge of Ω on a circle in $\textbf{L}^N \cup \mathbf {\Gamma }$ there are precisely four elements of $\mathbf {\Omega }^N$ (including Ω itself) sharing that edge; and
(vi) The symmetry group of Ω is the order-2 group
  $$\begin{aligned} \{ \textsf{R} \in O(4) \; : \; \textsf{R}\Omega =\Omega \} = \langle {\underline{\textsf{R}}}_{L_\Omega } \rangle , \end{aligned}$$
  where $L_\Omega$, the axis of Ω, is the great circle intersecting the midpoint of *C* and the centroid of $P_\Omega$.

For each $C \in \textbf{C}$ and for each $\Omega \in \mathbf {\Omega }^N_C$ we define the center of Ω to be the midpoint of $L_\Omega \cap \Omega$. Then the collection of centers of the $\Omega \in \mathbf {\Omega }^N_{C_{(1)}}$ is

4.22 $$\begin{aligned} \begin{aligned} \boldsymbol{\omega }^N_{C_{(1)}} := \{\omega ^N_{j\ell }\}_{j,\ell \in \mathbb {Z}}, \\ \omega ^N_{j\ell } := e^{{\textrm{i}}\pi \frac{2j+1}{2N}} \left( \cos \frac{\pi }{16}, ~ e^{{\textrm{i}}\pi \frac{2\ell +1}{4}} \sin \frac{\pi }{16} \right) , \end{aligned} \end{aligned}$$

and, for any given $j,\ell \in \mathbb {Z}$, we write $\Omega ^N_{j\ell }$ for the unique element of $\mathbf {\Omega }^N$ with center $\omega ^N_{j\ell }$.

### **Symmetries**

We continue to assume $N \in 4\mathbb {Z}$ positive, and we define the group

4.23 $$\begin{aligned} \mathscr {G}^N := \langle {\underline{\textsf{R}}}_\gamma \; : \; \gamma \in \textbf{L}^N \cup \mathbf {\Gamma }\rangle . \end{aligned}$$

#### Proposition 4.24

Let $N \in 4\mathbb {Z}$ be positive. Then $\mathscr {G}^N$ preserves each of $\textbf{C}$, $\{\mathbb {T}_i\}_{i=1}^3$, $\{T_C\}_{C \in \textbf{C}}$, $\textbf{L}^N$, $\textbf{p}^N$, $\mathbf {\Sigma }^N$, and $\mathbf {\Omega }^N$. Moreover, $\mathbf {\Omega }^N$ has at most two orbits under $\mathscr {G}^N$.

#### Proof

Granting that $\mathscr {G}^N$ preserves $\mathbf {\Omega }^N$, the final claim is clear from the evident facts that $\langle {\underline{\textsf{R}}}_L \;: \; L \in \bigcup _{i=2}^3 \textbf{L}^N_{\mathbb {T}_i} \rangle$ acts transitively on $\{\omega ^N_{j\ell }\}_{j+\ell \in 2\mathbb {Z}}$ and that $\langle {\underline{\textsf{R}}}_\Gamma \;: \; \Gamma \in \mathbf {\Gamma }\rangle$ acts transitively on $\{T_C\}_{C \in \textbf{C}}$. To check the remaining claims it suffices to do so at the level of the generators.

First suppose $L \in \textbf{L}^N$. Then $L \subset \mathbb {T}_i$ for some $i \in \{1,2,3\}$, and ${\underline{\textsf{R}}}_L$ clearly preserves $\mathbb {T}_i$, $\textbf{L}^N_{\mathbb {T}_i}$, and each $C \in \textbf{C}$ on $\mathbb {T}_i$ along with the corresponding $\textbf{p}^N_{C}$. Since ${\underline{\textsf{R}}}_L$ preserves $\mathbb {T}_i$ and each $C \in \textbf{C}$ on $\mathbb {T}_i$, it in fact preserves $\{\mathbb {T}_i\}_{i=1}^3$ and $\textbf{C}$. Since ${\underline{\textsf{R}}}_L$ preserves $\textbf{L}^N_{\mathbb {T}_i}$ and $\textbf{p}^N_{C}$ for each $C \in \textbf{C}$ on $\mathbb {T}_i$, it in fact preserves all of $\textbf{L}^N$ and all of $\textbf{p}^N$.

Now suppose $\Gamma \in \mathbf {\Gamma }$. Then Γ lies on some $\mathbb {T}_j$ and is equidistant from a pair of circles in $\textbf{C}$ on $\mathbb {T}_j$. In particular ${\underline{\textsf{R}}}_\Gamma$ preserves $\mathbb {T}_j$, $\textbf{L}^N_{\mathbb {T}_j}$, and the collection of those circles in $\textbf{C}$ on $\mathbb {T}_j$. It then follows easily that ${\underline{\textsf{R}}}_\Gamma$ preserves $\{\mathbb {T}_i\}_{i=1}^3$, $\textbf{C}$, all of $\textbf{L}^N$, and all of $\textbf{p}^N$.

Since ${\underline{\textsf{R}}}_L$ and ${\underline{\textsf{R}}}_\Gamma$ preserve $\textbf{C}$, they also preserve $\{T_C\}_{C \in \textbf{C}}$. Since they preserve $\textbf{p}^N$, they also preserve $\mathbf {\Sigma }^N$. Since they preserve $\{T_C\}_{C \in \textbf{C}}$, $\mathbf {\Sigma }^N$, and $\{\mathbb {T}_i\}_{i=1}^3$, they preserve $\mathbf {\Omega }^N$ too. □

For the reader’s reference we record the generators originating from $\mathbf {\Gamma }$:

4.25 $$\begin{aligned} \begin{aligned} {\underline{\textsf{R}}}_{\Gamma _{12}} \begin{bmatrix}w_1 \\ w_2\end{bmatrix} = \frac{1}{\sqrt{2}} \begin{bmatrix} 1 &  -{\textrm{i}}\\ {\textrm{i}}&  -1 \end{bmatrix} \begin{bmatrix}w_1 \\ w_2\end{bmatrix}, \qquad {\underline{\textsf{R}}}_{\Gamma _{1^\perp 2^\perp }} \begin{bmatrix}w_1 \\ w_2\end{bmatrix} = \frac{1}{\sqrt{2}} \begin{bmatrix} -1 &  {\textrm{i}}\\ -{\textrm{i}}&  1 \end{bmatrix} \begin{bmatrix}w_1 \\ w_2\end{bmatrix}, \\ {\underline{\textsf{R}}}_{\Gamma _{12^\perp }} \begin{bmatrix}w_1 \\ w_2\end{bmatrix} = \frac{1}{\sqrt{2}} \begin{bmatrix} 1 &  {\textrm{i}}\\ -{\textrm{i}}&  -1 \end{bmatrix} \begin{bmatrix}w_1 \\ w_2\end{bmatrix}, \qquad {\underline{\textsf{R}}}_{\Gamma _{1^\perp 2}} \begin{bmatrix}w_1 \\ w_2\end{bmatrix} = \frac{1}{\sqrt{2}} \begin{bmatrix} -1 &  -{\textrm{i}}\\ {\textrm{i}}&  1 \end{bmatrix} \begin{bmatrix}w_1 \\ w_2\end{bmatrix}, \\ {\underline{\textsf{R}}}_{\Gamma _{13}} \begin{bmatrix}w_1 \\ w_2\end{bmatrix} = \frac{1}{\sqrt{2}} \begin{bmatrix} 1 &  1 \\ 1 &  -1 \end{bmatrix} \begin{bmatrix}w_1 \\ w_2\end{bmatrix}, \qquad {\underline{\textsf{R}}}_{\Gamma _{1^\perp 3^\perp }} \begin{bmatrix}w_1 \\ w_2\end{bmatrix} = \frac{1}{\sqrt{2}} \begin{bmatrix} -1 &  -1 \\ -1 &  1 \end{bmatrix} \begin{bmatrix}w_1 \\ w_2\end{bmatrix}, \\ {\underline{\textsf{R}}}_{\Gamma _{13^\perp }} \begin{bmatrix}w_1 \\ w_2\end{bmatrix} = \frac{1}{\sqrt{2}} \begin{bmatrix} 1 &  -1 \\ -1 &  -1 \end{bmatrix} \begin{bmatrix}w_1 \\ w_2\end{bmatrix}, \qquad {\underline{\textsf{R}}}_{\Gamma _{1^\perp 3}} \begin{bmatrix}w_1 \\ w_2\end{bmatrix} = \frac{1}{\sqrt{2}} \begin{bmatrix} -1 &  1 \\ 1 &  1 \end{bmatrix} \begin{bmatrix}w_1 \\ w_2\end{bmatrix}, \\ {\underline{\textsf{R}}}_{\Gamma _{23}} \begin{bmatrix}w_1 \\ w_2\end{bmatrix} = \begin{bmatrix} 0 &  e^{-{\textrm{i}}\pi /4} \\ e^{{\textrm{i}}\pi /4} &  0 \end{bmatrix} \begin{bmatrix}w_1 \\ w_2\end{bmatrix}, \\ {\underline{\textsf{R}}}_{\Gamma _{2^\perp 3^\perp }} \begin{bmatrix}w_1 \\ w_2\end{bmatrix} = \begin{bmatrix} 0 &  -e^{-{\textrm{i}}\pi /4} \\ -e^{{\textrm{i}}\pi /4} &  0 \end{bmatrix} \begin{bmatrix}w_1 \\ w_2\end{bmatrix}, \\ {\underline{\textsf{R}}}_{\Gamma _{23^\perp }} \begin{bmatrix}w_1 \\ w_2\end{bmatrix} = \begin{bmatrix} 0 &  -e^{{\textrm{i}}\pi /4} \\ -e^{-{\textrm{i}}\pi /4} &  0 \end{bmatrix} \begin{bmatrix}w_1 \\ w_2\end{bmatrix}, \\ {\underline{\textsf{R}}}_{\Gamma _{2^\perp 3}} \begin{bmatrix}w_1 \\ w_2\end{bmatrix} = \begin{bmatrix} 0 &  e^{{\textrm{i}}\pi /4} \\ e^{-{\textrm{i}}\pi /4} &  0 \end{bmatrix} \begin{bmatrix}w_1 \\ w_2\end{bmatrix}. \end{aligned} \end{aligned}$$

### **Colorings**

By a coloring of $\mathbf {\Omega }^N$ we mean a function $c: \mathbf {\Omega }^N \rightarrow \{0,1\}$. Given any $\gamma \in \textbf{L}^N \cup \mathbf {\Gamma }$, we say that a coloring *c* is consistent along γ if for any $\Omega _1,\Omega _2 \in \mathbf {\Omega }^N$ sharing an edge on a circle $\gamma \in \mathbf {\Gamma }\cup \textbf{L}^N$ we have

4.26 $$\begin{aligned} c(\Omega _1)=c(\Omega _2) \; \Leftrightarrow \; {\underline{\textsf{R}}}_\gamma (\Omega _1)=\Omega _2. \end{aligned}$$

(Recall from 4.21.v that exactly four members of $\mathbf {\Omega }^N$ meet along a given edge on a given $\gamma \in \mathbf {\Gamma }\cup \textbf{L}^N$, and by 4.24 these four are exchanged in pairs under ${\underline{\textsf{R}}}_\gamma$.) We call *c* consistent if it is consistent along every $\gamma \in \textbf{L}^N \cup \mathbf {\Gamma }$. Note that a consistent coloring, if one exists, is uniquely determined by its value on any single member of $\mathbf {\Omega }^N$; thus, if there is one consistent coloring, then there are precisely two. Note finally that if $\textsf{R}\in O(4)$ preserves $\mathbf {\Omega }^N$ and *c* is a consistent coloring of $\mathbf {\Omega }^N$, then $c \circ \textsf{R}$ is also a consistent coloring of $\mathbf {\Omega }^N$.

#### Proposition 4.27

Let $N \in 4\mathbb {Z}$ be positive. The collection $\mathbf {\Omega }^N$ admits a consistent coloring *c* if and only if $\mathbf {\Omega }^N$ has precisely two orbits under $\mathscr {G}^N$, namely the preimages under *c* of 0 and 1.

#### Proof

Suppose *c* is a consistent coloring of $\mathbf {\Omega }^N$. We observed in 4.24 that $\mathbf {\Omega }^N$ has at most two orbits under $\mathscr {G}^N$, and it is clear that for each $\chi \in \{0,1\}$ there is a single orbit containing $c^{-1}(\{\chi \})$. To complete the proof in this direction (⇒) it suffices to take any $\gamma \in \textbf{L}^N \cup \mathbf {\Gamma }$ and check that ${\underline{\textsf{R}}}_\gamma$ preserves *c*, but $c \circ {\underline{\textsf{R}}}_\gamma$ is a consistent coloring which agrees with *c* on all prisms having an edge on γ and therefore indeed agrees with *c* globally. The converse direction, which we will not need, follows even more directly from the definitions. □

#### Proposition 4.28

Let $N \in 4+8\mathbb {Z}$ be positive. Then $\mathbf {\Omega }^N$ admits a consistent coloring.

#### Proof

We define a coloring *c* and confirm its consistency. First we define $c|_{\mathbf {\Omega }^N_{C_{(1)}}}$ by

4.29 $$\begin{aligned} c(\Omega ^N_{j\ell }) = (j+\ell ) \bmod 2. \end{aligned}$$

Next, for each $i \in \{2,3,2^\perp ,3^\perp \}$ we set

4.30 $$\begin{aligned} c|_{\mathbf {\Omega }^N_{C_{(j)}}} = c|_{\mathbf {\Omega }^N_{C_{(1)}}} \circ {\underline{\textsf{R}}}_{\Gamma _{1j}}. \end{aligned}$$

We complete the definition of *c* by setting

4.31 $$\begin{aligned} c|_{\mathbf {\Omega }^N_{C_{(1)}^\perp }} = c|_{\mathbf {\Omega }^N_{C_{(3)}}} \circ {\underline{\textsf{R}}}_{\Gamma _{1^\perp 3}}. \end{aligned}$$

It is obvious from the construction of *c* that it is consistent along each circle in

4.32 $$\begin{aligned} \textbf{L}^N \cup \{ \Gamma _{12}, \Gamma _{12^\perp }, \Gamma _{13}, \Gamma _{13^\perp }, \Gamma _{1^\perp 3} \}, \end{aligned}$$

so it remains to check consistency along the seven circles

4.33 $$\begin{aligned} \Gamma _{23}, \Gamma _{23^\perp }, \Gamma _{2^\perp 3},\Gamma _{2^\perp 3^\perp }, \Gamma _{1^\perp 2}, \Gamma _{1^\perp 2^\perp }, \Gamma _{1^\perp 3^\perp }. \end{aligned}$$

For $i \in \{2,2^\perp \}$ and $j \in \{3,3^\perp \}$ to verify consistency along $\Gamma _{ij}$ (accounting for the first four circles in this last list) it suffices to check that

4.34 $$\begin{aligned} {\underline{\textsf{R}}}_{\Gamma _{1j}} {\underline{\textsf{R}}}_{\Gamma _{ij}} {\underline{\textsf{R}}}_{\Gamma _{1i}} \omega ^N_{00} \in \{\omega ^N_{k\ell }\}_{k+\ell \in 2\mathbb {Z}} \quad (i \in \{2,2^\perp \}, j \in \{3,3^\perp \}), \end{aligned}$$

while for $j \in \{2,2^\perp ,3^\perp \}$ to verify consistency along $\Gamma _{1^\perp j}$ (accounting for the final three circles) it suffices to check that

4.35 $$\begin{aligned} {\underline{\textsf{R}}}_{\Gamma _{1j}} {\underline{\textsf{R}}}_{\Gamma _{1^\perp j}} {\underline{\textsf{R}}}_{\Gamma _{1^\perp 3}} {\underline{\textsf{R}}}_{\Gamma _{13}}\omega ^N_{00} \in \{\omega ^N_{k\ell }\}_{k+\ell \in 2\mathbb {Z}} \quad (j \in \{2,2^\perp ,3^\perp \}). \end{aligned}$$

We compute

4.36 $$\begin{aligned} \begin{aligned} {\underline{\textsf{R}}}_{\Gamma _{12}} {\underline{\textsf{R}}}_{\Gamma _{1^\perp 2}} {\underline{\textsf{R}}}_{\Gamma _{1^\perp 3}} {\underline{\textsf{R}}}_{\Gamma _{13}} \begin{bmatrix}w_1\\ w_2\end{bmatrix} = \begin{bmatrix} -{\textrm{i}}&  0 \\ 0 &  {\textrm{i}}\end{bmatrix} \begin{bmatrix}w_1\\ w_2\end{bmatrix}, \\ {\underline{\textsf{R}}}_{\Gamma _{12^\perp }} {\underline{\textsf{R}}}_{\Gamma _{1^\perp 2^\perp }} {\underline{\textsf{R}}}_{\Gamma _{1^\perp 3}} {\underline{\textsf{R}}}_{\Gamma _{13}} \begin{bmatrix}w_1\\ w_2\end{bmatrix} = \begin{bmatrix} {\textrm{i}}&  0 \\ 0 &  -{\textrm{i}}\end{bmatrix} \begin{bmatrix}w_1\\ w_2\end{bmatrix}, \\ {\underline{\textsf{R}}}_{\Gamma _{13^\perp }} {\underline{\textsf{R}}}_{\Gamma _{1^\perp 3^\perp }} {\underline{\textsf{R}}}_{\Gamma _{1^\perp 3}} {\underline{\textsf{R}}}_{\Gamma _{13}} \begin{bmatrix}w_1\\ w_2\end{bmatrix} = \begin{bmatrix} -1 &  0 \\ 0 &  -1 \end{bmatrix} \begin{bmatrix}w_1\\ w_2\end{bmatrix}, \end{aligned} \end{aligned}$$

whence follows 4.35, using the fact that $N \in 4\mathbb {Z}$, and we compute

4.37 $$\begin{aligned} \begin{aligned} {\underline{\textsf{R}}}_{\Gamma _{13}}{\underline{\textsf{R}}}_{\Gamma _{23}}{\underline{\textsf{R}}}_{\Gamma _{12}} \begin{bmatrix}w_1\\ w_2\end{bmatrix} = {\underline{\textsf{R}}}_{\Gamma _{13^\perp }} {\underline{\textsf{R}}}_{\Gamma _{2^\perp 3^\perp }} {\underline{\textsf{R}}}_{\Gamma _{12^\perp }} \begin{bmatrix}w_1\\ w_2\end{bmatrix} = \begin{bmatrix} e^{{\textrm{i}}\pi /4} &  0 \\ 0 &  -e^{-{\textrm{i}}\pi /4} \end{bmatrix} \begin{bmatrix}w_1\\ w_2\end{bmatrix}, \\ {\underline{\textsf{R}}}_{\Gamma _{13^\perp }} {\underline{\textsf{R}}}_{\Gamma _{23^\perp }}{\underline{\textsf{R}}}_{\Gamma _{12}} \begin{bmatrix}w_1\\ w_2\end{bmatrix} = {\underline{\textsf{R}}}_{\Gamma _{13}} {\underline{\textsf{R}}}_{\Gamma _{2^\perp 3}}{\underline{\textsf{R}}}_{\Gamma _{12^\perp }} \begin{bmatrix}w_1\\ w_2\end{bmatrix} = \begin{bmatrix} e^{-{\textrm{i}}\pi /4} &  0 \\ 0 &  -e^{{\textrm{i}}\pi /4} \end{bmatrix} \begin{bmatrix}w_1\\ w_2\end{bmatrix}, \end{aligned} \end{aligned}$$

whence follows 4.34, using the assumption that $N \in 4+8\mathbb {Z}$. □

We can now describe $\mathscr {G}^N$ in more detail. Items i and ii below are important for the construction, and we mention items ix and x because they are helpful in relating the surfaces constructed here with certain surfaces constructed in [11]; we include the remaining items simply to offer a fuller picture of $\mathscr {G}^N$.

#### Proposition 4.38

Let $N \in 4+8\mathbb {Z}$ be positive. Then

(i) The action of $\mathscr {G}^N$ on $\mathbf {\Omega }^N$ has precisely two orbits;
(ii) For each $\Omega \in \mathbf {\Omega }^N$ we have ${\underline{\textsf{R}}}_{L_\Omega } \in \mathscr {G}^N$ (recalling 4.21.vi);
(iii) $\mathscr {G}^N$ has order 48*N*;
(iv) The group homomorphism $\widehat{\Pi }_{\textrm{Hopf}}|_{\mathscr {G}^N} \rightarrow O(3)$ (recalling 2.7 and 2.8) has kernel the cyclic group
  $$\begin{aligned} \ker \widehat{\Pi }_{\textrm{Hopf}}|_{\mathscr {G}^N} = \mathbb {Z}_N := \left\langle \textsf{R}_{C_{(1)}}^{2\pi /N} \textsf{R}_{C_{(1)}^\perp }^{2\pi /N} \right\rangle \end{aligned}$$
  and image the octahedral group (of order 48)
  $$\begin{aligned} \operatorname {im}\widehat{\Pi }_{\textrm{Hopf}}|_{\mathscr {G}^N} = O_h := \{ \textsf{r} \in O(3) \; : \; \textsf{r} ([-1,1]^3) =[-1,1]^3 \}. \end{aligned}$$
(v) The stabilizer $\operatorname {Stab}_{\mathscr {G}^N}(T_{C_{(1)}}):= \{ \textsf{R}\in \mathscr {G}^N \;: \; \textsf{R}T_{C_{(1)}} = T_{C_{(1)}} \}$ of $T_{C_{(1)}}$ in $\mathscr {G}^N$ has order 8*N* and is generated by ${\underline{\textsf{R}}}_{L_{\Omega _{00}}}$, ${\underline{\textsf{R}}}_{L_2}$, and ${\underline{\textsf{R}}}_{L_3}$ for any single $L_2 \in \textbf{L}^N_{\mathbb {T}_2}$ and any single $L_3 \in \textbf{L}^N_{\mathbb {T}_3}$;
(vi) $\mathscr {G}^N$ can be obtained from $\operatorname {Stab}_{\mathscr {G}^N}(T_{C_{(1)}})$ by adjoining ${\underline{\textsf{R}}}_{\Gamma _{12}}$;
(vii) The subgroup $\mathscr {G}_{\textbf{L}^N}:= \langle {\underline{\textsf{R}}}_L \rangle _{L \in \textbf{L}^N}$ of $\mathscr {G}^N$ has order 8*N*;
(viii) The subgroup $\mathscr {G}_\Gamma := \langle {\underline{\textsf{R}}}_\Gamma \rangle _{\Gamma \in \mathbf {\Gamma }}$ of $\mathscr {G}^N$ has order 48;
(ix) $$ \textsf{R}_{C_{(1)}}^{\pi /2} \left( \textsf{R}_{C_{(1)}}^{\pi /N} \textsf{R}_{C_{(1)}^\perp }^{\pi /N} \right) \in \mathscr {G}^N $$; and
(x) For any $L \in \textbf{L}^N$ we have $\textsf{R}_L^{\pi /4}\textsf{R}_{L^\perp }^{\pi /4} \in \mathscr {G}^N$, where the relative orientation of *L* and $L^\perp$ is the one such that this product preserves the $\mathbb {T}_i$ containing $L \cup L^\perp$.

#### Proof

Item i is immediate from 4.27 and 4.28. Given that ${\underline{\textsf{R}}}_L \in \mathscr {G}^N$ for every $L \in \textbf{L}^N$, item x is equivalent to

4.39 $$\begin{aligned} \textsf{R}_{C_{(1)}}^{\frac{\pi }{N}+\frac{\pi }{2}} \textsf{R}_{C_{(1)}^\perp }^{\frac{\pi }{N}} \in \mathscr {G}^N, \end{aligned}$$

which is in fact identical to claim ix. However, it is clear that

4.40 $$\begin{aligned} \textsf{R}_{C_{(1)}}^{2\pi /N}\textsf{R}_{C_{(1)}^\perp }^{2\pi /N} \in \langle {\underline{\textsf{R}}}_L \rangle _{L \in \textbf{L}^N_{\mathbb {T}_2}} < \mathscr {G}^N, \end{aligned}$$

and we see from 4.37 that

4.41 $$\begin{aligned} \textsf{R}_{C_{(1)}}^{\pi /4}\textsf{R}_{C_{(1)}^\perp }^{-\pi /4} \in \mathscr {G}^N, \end{aligned}$$

so, because our assumption on *N* ensures that $\frac{N+4}{8}$ is a positive integer, we also have

4.42 $$\begin{aligned} \textsf{R}_{C_{(1)}}^{\frac{\pi }{N}+\frac{\pi }{2}} \textsf{R}_{C_{(1)}^\perp }^{\frac{\pi }{N}} = \textsf{R}_{C_{(1)}}^{\pi /4}\textsf{R}_{C_{(1)}^\perp }^{-\pi /4} \left( \textsf{R}_{C_{(1)}}^{\frac{2\pi }{N}} \textsf{R}_{C_{(1)}^\perp }^{\frac{2\pi }{N}} \right) ^{\frac{N+4}{8}} \in \mathscr {G}^N, \end{aligned}$$

verifying 4.39, and so items ii and ix.

In view of item i (and the fact that $\mathbf {\Omega }^N$ has cardinality 48*N*) the $\mathscr {G}^N$ orbit of any $\Omega \in \mathbf {\Omega }^N$ consists of 24*N* prisms, while item ii and 4.21.vi imply that the stabilizer in $\mathscr {G}^N$ of any $\Omega \in \mathbf {\Omega }^N$ has order two, confirming item iii.

For iv it is clear that each ${\underline{\textsf{R}}}_\gamma$ with $\gamma \in \textbf{L}^N \cup \mathbf {\Gamma }$ preserves the set of fibers of $\Pi _{\textrm{Hopf}}$, and so the map in the statement is indeed well-defined. Since $\mathscr {G}^N$ preserves $\textbf{C}$, it is also clear that $\widehat{\Pi }_{\textrm{Hopf}}(\mathscr {G}^N)$ is a subgroup of $O_h$. Investigating more closely, we observe that $\widehat{\Pi }_{\textrm{Hopf}}(\{{\underline{\textsf{R}}}_\Gamma \}_{\Gamma \in \mathbf {\Gamma }})$ consists of the six reflections through each of the six lines that pass through the origin and a pair of midpoints of edges of the cube $[-1,1]^3$. These last six symmetries generate $O_h \cap SO(3)$, and so by adjoining any single $\widehat{\Pi }_{\textrm{Hopf}}({\underline{\textsf{R}}}_L)$ with $L \in \textbf{L}^N$ we obtain the full group $O_h$, verifying the assertion about the image of the map in iv. On the other hand, from 4.40 we see that the kernel contains $\mathbb {Z}_N$, but the orders of $\mathbb {Z}_N$, $\mathscr {G}^N$, and $O_h$ are respectively *N*, 48*N*, and 48, completing the proof of iv.

We proceed to item v. Using item i again, we can verify that the stabilizer of $T_{C_{(1)}}$ in $\mathscr {G}^N$ acting on $\mathbf {\Omega }^N_{C_{(1)}}$ has two orbits, each of cardinality 4*N*. On the other hand, for any $\Omega \in \mathbf {\Omega }^N_{C_{(1)}}$ the reflection ${\underline{\textsf{R}}}_{L_\Omega }$ preserves $T_{C_{(1)}}$, so with item ii and 4.21.vi, we verify the asserted order in item v. The proof of v is then completed by observing that, for any $L_2,L_3$ as in the second half of the statement of v, the group $\langle {\underline{\textsf{R}}}_{L_{\Omega _{00}}}, {\underline{\textsf{R}}}_{L_2}, {\underline{\textsf{R}}}_{L_3} \rangle$ also has order 8*N* and preserves $T_{C_{(1)}}$.

Now, for vi, let $\mathscr {H}$ be the group generated by the ${\underline{\textsf{R}}}_{\Gamma _{12}}$ and the stabilizer of $T_{C_{(1)}}$ in $\mathscr {G}^N$. To prove vi it suffices to check that $\mathscr {H}$ includes ${\underline{\textsf{R}}}_\gamma$ for every $\gamma \in \Gamma \cup \textbf{L}^N$. Clearly ${\underline{\textsf{R}}}_L \in \mathscr {H}$ for every $L \in \textbf{L}^N_{\mathbb {T}_2} \cup \textbf{L}^N_{\mathbb {T}_3}$, but ${\underline{\textsf{R}}}_{\Gamma _{12}}\textbf{L}^N_{\mathbb {T}_2}=\textbf{L}^N_{\mathbb {T}_1}$, so indeed $\langle {\underline{\textsf{R}}}_L \rangle _{L \in \textbf{L}^N} < \mathscr {H}$. Since ${\underline{\textsf{R}}}_{C_{(1)}} \in \langle {\underline{\textsf{R}}}_L \rangle _{L \in \textbf{L}^N_{\mathbb {T}_2} \cup \textbf{L}^N_{\mathbb {T}_3}} < \mathscr {H}$ and ${\underline{\textsf{R}}}_{\Gamma _{12}} \in \mathscr {H}$, we get also ${\underline{\textsf{R}}}_{\Gamma _{12\perp }}, ~ {\underline{\textsf{R}}}_{C_{(2)}} \in \mathscr {H}$, and then similarly ${\underline{\textsf{R}}}_{\Gamma _{1^\perp 2}}, ~ {\underline{\textsf{R}}}_{C_{(1)}^\perp }, ~ {\underline{\textsf{R}}}_{C_{(2)}^\perp }, ~ {\underline{\textsf{R}}}_{\Gamma _{1^\perp 2^\perp }} \in \mathscr {H}$. In particular we have ${\underline{\textsf{R}}}_\Gamma \in \mathscr {H}$ for every $\Gamma \in \mathbf {\Gamma }$ such that $\Gamma \subset \mathbb {T}_3$. Using ${\underline{\textsf{R}}}_{L_{\Omega _{00}}} \in \mathscr {H}$, ${\underline{\textsf{R}}}_{L_{\Omega _{00}}}\mathbb {T}_3 = \mathbb {T}_2$, and ${\underline{\textsf{R}}}_{L_{\Omega _{00}}}\mathbb {T}_1 = \mathbb {T}_1$, we then get ${\underline{\textsf{R}}}_\Gamma \in \mathscr {H}$ for every $\Gamma \in \mathbf {\Gamma }$ such that $\Gamma \subset \mathbb {T}_2$. Finally, since ${\underline{\textsf{R}}}_{\Gamma _{12}}$ preserves $\mathbb {T}_3$ and exchanges $\mathbb {T}_1$ and $\mathbb {T}_2$, we get in turn ${\underline{\textsf{R}}}_\Gamma \in \mathscr {H}$ for every $\Gamma \in \mathbf {\Gamma }$ such that $\Gamma \subset \mathbb {T}_1$, completing the proof of vi.

Turning to vii, it is easy to see that the orbit under $\mathscr {G}_{\textbf{L}^N}$ of any $\Omega \in \mathbf {\Omega }^N_{C_{(1)}}$ includes at least 4*N* members of $\mathbf {\Omega }^N_{C_{(1)}}$ and at least 4*N* members of $\mathbf {\Omega }^N_{C_{(1)}^\perp }$. In view of item i this orbit does not contain any other members of $\mathbf {\Omega }^N_{C_{(1)}} \cup \mathbf {\Omega }^N_{C_{(1)}^\perp }$, and since each ${\underline{\textsf{R}}}_L$ with $L \in \textbf{L}^N$ preserves each $\mathbb {T}_i$, the orbit of Ω under $\mathscr {G}_{\textbf{L}^N}$ in fact consists of exactly these 8*N* prisms and $\mathscr {G}_{\textbf{L}^N}$ does not include $L_\Omega$, ending the proof of vii.

One can give a proof of viii similar to that of 4.28. In outline, first we observe that every ${\underline{\textsf{R}}}_\Gamma$ with $\Gamma \in \mathbf {\Gamma }$ is unitary (preserves the orientations on the fibers of $\Pi _{\textrm{Hopf}}$), while ${\underline{\textsf{R}}}_{L_{\Omega _{00}}}$ is antiunitary (reverses the orientations on the fibers of $\Pi _{\textrm{Hopf}}$), and so it suffices to check that the orbit of $\Omega _{00}$ under $\mathscr {G}_\Gamma$ has cardinality 48. We may assume (in this paragraph) N=4. Next we define a map $c: \mathbf {\Omega }^4 \rightarrow \{0,1,2,3\}$ by first defining $c|_{\mathbf {\Omega }^4_{C_{(1)}}}$ as $c(\Omega _{ij}):= (j-i) \bmod 4$ and then extending to all of $\mathbf {\Omega }^4$ as in the proof of 4.28. Then one can check, much as in the proof of 4.28 and with some modifications to the discussion immediately preceding it, that $\mathscr {G}_\Gamma$ preserves *c* and then that it has order 48. We leave the details to the reader.

It remains only to check item x. In fact it suffices to consider $L \in \textbf{L}^N_{\mathbb {T}_3}$. Then *L* and $L^\perp$ orthogonally intersect both $C_{(1)}$ and $\Gamma _{12}$; furthermore $C_{(1)}, \Gamma _{12} \subset \mathbb {T}_3$, and $d^{\mathbb {S}^3}_{C_{(1)}}$ takes the constant value $\frac{\pi }{8}$ on $\Gamma _{12}$. Accordingly,

4.43 $$\begin{aligned} \textsf{R}_{L}^{\pi /4}\textsf{R}_{L^\perp }^{\pi /4} = {\underline{\textsf{R}}}_{\Gamma _{12}}{\underline{\textsf{R}}}_{C_{(1)}} \in \mathscr {G}^N, \end{aligned}$$

where each of *L* and $L^\perp$ is oriented from either point of intersection with $C_{(1)}$ toward the closest point (to this one) of intersection with $\Gamma _{12}$, completing the proof. □

### **Proof of Theorem**1.1

Let N=4+8m, and let *Q* be the hexagon in $\partial \Omega _{00}$ which is the union of the edges contained in $\bigcup \mathbf {\Gamma }\cup \bigcup \textbf{L}^N$. By 4.21.i and 3.25 there exists a unique connected minimal surface $D \subset \Omega$ with $\partial \Omega =Q$, and *D* is an embedded disc. By the maximum principle $D \cap \partial \Omega _{00} = \partial D$. Then from the uniqueness of *D* and the fact (4.38.i) that the action of $\mathscr {G}^N$ on $\mathbf {\Omega }^N$ has two orbits it follows that the set $M_m:= \mathscr {G}^N(D)$ is a closed embedded minimal surface.

From the geometry of $\Omega _{00}$ (4.21 and 3.8) we see that every vertex angle of *Q* is right. Using the Gauss-Bonnet theorem and the fact that *D* is a disc with piecewise geodesic boundary, we conclude that *D* has total curvature -π. Thus $M_m$ has total curvature -24Nπ, and another application of the Gauss-Bonnet theorem then establishes that $M_m$ has genus 6N+1=48m+25. By construction $M_m$ contains $\bigcup \mathbf {\Gamma }\cup \textbf{L}^N$ and is invariant under $\mathscr {G}^N$. This completes the proof of the existence portion of 1.1.

The uniqueness assertion follows immediately from the reflection principle and 3.25. To prove that $M_m$ is congruent, for sufficiently large *m*, to the surface constructed in [11, Theorem 7.1] as a small graphical perturbation of the initial surface N(2,2m+1,1,1,1,0,1) specified in [11, Definition 4.13] it suffices—as explained at the end of the proof of Sect. 3, in the proof of Theorem 1.2—to verify that (modulo congruence) this initial surface is invariant under $\mathscr {G}^N$ and that its intersection with the interior of $\Omega _{00}$ is nonempty and has boundary ∂D.

These last properties are easily checked by inspection of [11, 4.15] (reviewing also the supporting definitions), with the aid of 4.38.ix and 4.38.x. For this it is important to note an isolated misprint in [11, 4.15]: the $\mathbb {S}^3$ isometry

4.44 $$\begin{aligned} \textsf{R}_{\underline{C}}^{\pi /kmn'_{(-1)^j}} \; \textsf{R}_{\underline{C}^\perp }^{\pi /kmn'_{(-1)^j}} \end{aligned}$$

appearing in parentheses in the middle line of [11, 4.15] is missing factors of 2 in the denominators of both exponents and should instead read

4.45 $$\begin{aligned} \textsf{R}_{\underline{C}}^{\pi /2kmn'_{(-1)^j}} \; \textsf{R}_{\underline{C}^\perp }^{\pi /2kmn'_{(-1)^j}}, \end{aligned}$$

which in the notation of the present article is (up to an overall congruence)

4.46 $$\begin{aligned} \textsf{R}_{C_{(1)}}^{\pi /N} \; \textsf{R}_{C_{(1)}^\perp }^{\pi /N}. \end{aligned}$$

The significance of this correction is that the isometry 4.44 amounts to shifting the corresponding singly periodic Scherk surface—glued in along the great circle indexed by *j*—by a full period, rather than a half period, counter to the intended effect of producing distinct initial surfaces, which is instead accomplished by 4.45, corresponding to a half-period shift.

## Data Availability

No datasets were generated or analysed during the current study.
